# Impact of Different Tidal Volume Levels at Low Mechanical Power on Ventilator-Induced Lung Injury in Rats

**DOI:** 10.3389/fphys.2018.00318

**Published:** 2018-04-04

**Authors:** Lillian Moraes, Pedro L. Silva, Alessandra Thompson, Cintia L. Santos, Raquel S. Santos, Marcos V. S. Fernandes, Marcelo M. Morales, Vanessa Martins, Vera L. Capelozzi, Marcelo G. de Abreu, Paolo Pelosi, Patricia R. M. Rocco

**Affiliations:** ^1^Laboratory of Pulmonary Investigation, Carlos Chagas Filho Institute of Biophysics, Federal University of Rio de Janeiro, Rio de Janeiro, Brazil; ^2^Laboratory of Cellular and Molecular Physiology, Carlos Chagas Filho Institute of Biophysics, Federal University of Rio de Janeiro, Rio de Janeiro, Brazil; ^3^Department of Pathology, School of Medicine, University of São Paulo, São Paulo, Brazil; ^4^Pulmonary Engineering Group, Department of Anesthesiology and Intensive Care Therapy, University Hospital Carl Gustav Carus, Dresden University of Technology, Dresden, Germany; ^5^Department of Surgical Sciences and Integrated Diagnostics, San Martino Policlinico Hospital, IRCCS for Oncology, University of Genoa, Genoa, Italy

**Keywords:** mechanical power, energy, extracellular matrix, driving pressure, respiratory rate, tidal volume

## Abstract

Tidal volume (V_T_) has been considered the main determinant of ventilator-induced lung injury (VILI). Recently, experimental studies have suggested that mechanical power transferred from the ventilator to the lungs is the promoter of VILI. We hypothesized that, as long as mechanical power is kept below a safe threshold, high V_T_ should not be injurious. The present study aimed to investigate the impact of different V_T_ levels and respiratory rates (RR) on lung function, diffuse alveolar damage (DAD), alveolar ultrastructure, and expression of genes related to inflammation [interleukin (IL)-6], alveolar stretch (amphiregulin), epithelial [club cell secretory protein (CC)16] and endothelial [intercellular adhesion molecule (ICAM)-1] cell injury, and extracellular matrix damage [syndecan-1, decorin, and metalloproteinase (MMP)-9] in experimental acute respiratory distress syndrome (ARDS) under low-power mechanical ventilation. Twenty-eight Wistar rats received *Escherichia coli* lipopolysaccharide intratracheally. After 24 h, 21 animals were randomly assigned to ventilation (2 h) with low mechanical power at three different V_T_ levels (*n* = 7/group): (1) V_T_ = 6 mL/kg and RR adjusted to normocapnia; (2) V_T_ = 13 mL/kg; and 3) V_T_ = 22 mL/kg. In the second and third groups, RR was adjusted to yield low mechanical power comparable to that of the first group. Mechanical power was calculated as [(Δ${\text{P},}_{\text{L}}^{2}$/Est,_L_)/2]× RR (ΔP,_L_ = transpulmonary driving pressure, Est,_L_ = static lung elastance). Seven rats were not mechanically ventilated (NV) and were used for molecular biology analysis. Mechanical power was comparable among groups, while V_T_ gradually increased. ΔP,_L_ and mechanical energy were higher in V_T_ = 22 mL/kg than V_T_ = 6 mL/kg and V_T_ = 13 mL/kg (*p* < 0.001 for both). Accordingly, DAD score increased in V_T_ = 22 mL/kg compared to V_T_ = 6 mL/kg and V_T_ = 13 mL/kg [23(18.5–24.75) vs. 16(12–17.75) and 16(13.25–18), *p* < 0.05, respectively]. V_T_ = 22 mL/kg was associated with higher IL-6, amphiregulin, CC16, MMP-9, and syndecan-1 mRNA expression and lower decorin expression than V_T_ = 6 mL/kg. Multiple linear regression analyses indicated that V_T_ was able to predict changes in IL-6 and CC16, whereas ΔP,_L_ predicted pHa, oxygenation, amphiregulin, and syndecan-1 expression. In the model of ARDS used herein, even at low mechanical power, high V_T_ resulted in VILI. V_T_ control seems to be more important than RR control to mitigate VILI.

## Introduction

Mechanical ventilation with high tidal volume (V_T_) can promote ventilator-induced lung injury (VILI) (Tremblay and Slutsky, 2006). In patients with the acute respiratory distress syndrome (ARDS), V_T_ level has been recognized as a major risk factor for organ failure and death (Brower et al., 2000; Putensen et al., 2009), and the use of low V_T_ (4–8 mL/kg) represents the cornerstone of protective mechanical ventilation (Bellani et al., 2016; Fan et al., 2017). Additionally, the respiratory system driving pressure (ΔP,_RS_) is associated with increased lung inflammation (Bellani et al., 2011) and predicts mortality rate in ARDS (Amato et al., 2015). Respiratory rate (RR) (Rich et al., 2003) as well as inspiratory (Fujita et al., 2007) and expiratory (Schumann et al., 2014) airflow have also been implicated in VILI. The knowledge that different respiratory variables may be injurious has led to the concept that mechanical energy or power transferred from the ventilator to the lungs may be determinants of VILI pathogenesis (Cressoni et al., 2016). According to this concept, we hypothesized that, as long as mechanical power is kept below a safe threshold, high V_T_ levels should not be injurious to the lungs.

Thus, the present study aimed to investigate the impact of different V_T_ levels and RR on lung function, diffuse alveolar damage (DAD), alveolar ultrastructure, and expression of genes related to inflammation [interleukin (IL)-6], alveolar stretch (amphiregulin), epithelial [club cell secretory protein (CC)16] and endothelial [intercellular adhesion molecule (ICAM)-1] cell injury, and extracellular matrix damage [syndecan-1, decorin, and metalloproteinase (MMP)-9] in experimental ARDS under low-power mechanical ventilation.

## Materials and methods

### Ethics statement

This study was approved by the Animal Care and Use Committee (CEUA: 064-17) of the Health Sciences Center, Federal University of Rio de Janeiro, Rio de Janeiro, Brazil (chair: Prof. M. Frajblat). All animals received humane care in compliance with the “Principles of Laboratory Animal Care” formulated by the National Society for Medical Research and the U.S. National Academy of Sciences *Guide for the Care and Use of Laboratory Animals*. The present study followed the ARRIVE guidelines for reporting of animal research (Kilkenny et al., 2010).

### Animal preparation and experimental protocol

Twenty-eight Wistar rats (weight 383 ± 80 g) were anaesthetized by inhalation of sevoflurane 2% (Sevorane®; Cristália, Itapira, SP, Brazil), and intratracheal (i.t.) instillation of *Escherichia coli* lipopolysaccharide (O55:B5, LPS Ultrapure, Invivogen), 200 μg suspended in saline solution to a total volume of 200 μL (Samary et al., 2016a) to induce acute lung inflammation. After 24 h, animals were premedicated intraperitoneally (i.p.) with 10 mg/kg diazepam (Compaz, Cristália, Itapira, SP, Brazil), followed by 100 mg/kg ketamine (Ketamin-S+, Cristália, Itapira, SP, Brazil) and 2 mg/kg midazolam (Dormicum, União Química, São Paulo, SP, Brazil). After local anesthesia with 2% lidocaine (0.4 mL), a midline neck incision and tracheostomy were performed. Seven rats were used for molecular biology analysis and were not mechanically ventilated (nonventilated, NV).

An intravenous (i.v.) catheter (Jelco 24G, Becton, Dickinson and Company, New Jersey, NJ, USA) was inserted into the tail vein, and anesthesia induced and maintained with midazolam (2 mg/kg/h) and ketamine (50 mg/kg/h). Additionally, 10 mL/kg/h Ringer's lactate (B. Braun, Crissier, Switzerland) was administered i.v. Gelafundin® 4% (B. Braun, São Gonçalo, RJ, Brazil) was administered intravenously (in 0.5-mL increments) to maintain mean arterial pressure (MAP) >60 mmHg. A second catheter (18G, Arrow International, USA) was then placed in the right internal carotid artery for blood sampling and gas analysis (Radiometer ABL80 FLEX, Copenhagen NV, Denmark), as well as monitoring of MAP (Networked Multiparameter Veterinary Monitor LifeWindow 6,000 V; Digicare Animal Health, Boynton Beach, FL, USA). A 30-cm-long water-filled catheter (PE-205, Becton, Dickinson and Company) with side holes at the tip, connected to a differential pressure transducer (UT-PL-400, SCIREQ, Montreal, QC, Canada), was used to measure the esophageal pressure (Pes). The catheter was passed into the stomach and then slowly returned into the esophagus; its proper positioning was assessed with the “occlusion test” (Baydur et al., 1982). Heart rate (HR), MAP, and rectal temperature were continuously monitored (Networked Multiparameter Veterinary Monitor LifeWindow 6,000V, Digicare Animal Health, Florida, USA). Body temperature was maintained at 37.5 ± 1°C using a heating bed.

**Figure 1:**
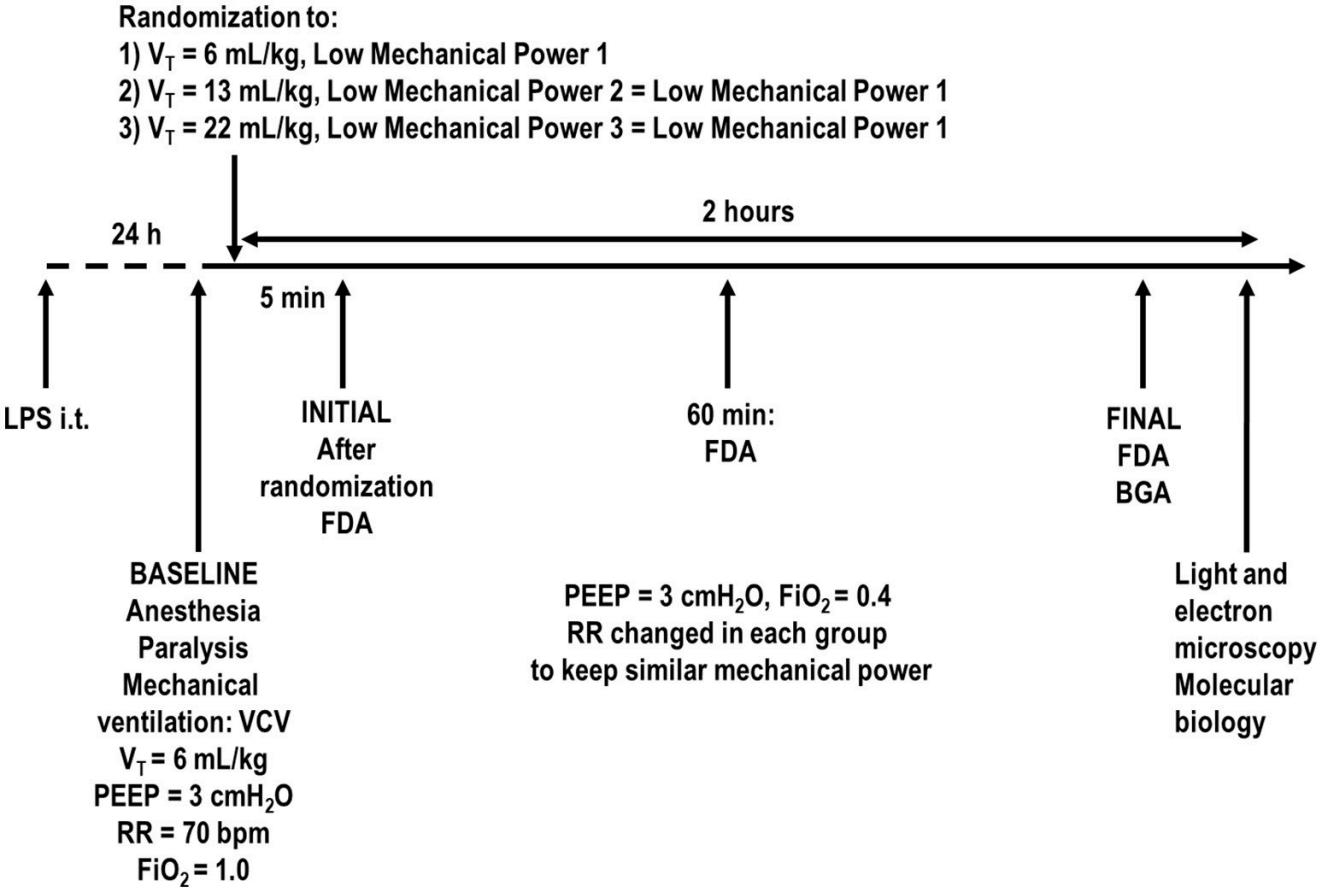
Timeline representation of the experimental protocol. FDA, functional data acquisition; BGA, blood gas analysis; FiO_2_, fraction of inspired oxygen; LPS, *Escherichia coli* lipopolysaccharide; i.t., intratracheally; PEEP, positive end-expiratory pressure; PV curve, pressure-volume curve; RR, respiratory rate; VCV, volume-controlled ventilation; V_T_, tidal volume. Lung mechanics were assessed every 15 min.

Animals were paralyzed with pancuronium bromide (2 mg/kg, i.v.), and their lungs mechanically ventilated (Servo-i, MAQUET, Solna, Sweden) in volume-controlled mode (VCV) with constant inspiratory airflow, V_T_ = 6 mL/kg, RR to maintain normocapnia (PaCO_2_ = 35–45 mmHg) (around 70 bpm), positive end-expiratory pressure (PEEP) = 3 cmH_2_O, FiO_2_ = 1.0, and an inspiratory-expiratory ratio of 1:2 (Figure 1). Arterial blood gases (300 μl) were determined using a Radiometer ABL80 FLEX analyzer (Copenhagen NV, Denmark) and respiratory system mechanics were assessed (BASELINE). FiO_2_ was reduced to 0.4 to prevent possible iatrogenic effects. Rats were then assigned to three different tidal volumes with low mechanical power (*n* = 7/group): (1) V_T_ = 6 mL/kg and RR adjusted to normocapnia; (2) V_T_ = 13 mL/kg; (3) V_T_ = 22 mL/kg. In the second and third groups, RR was adjusted to yield mechanical power comparable to that of the first group. The V_T_ level of 6 mL/kg combined with RR to lead to normocapnia (Samary et al., 2015) corresponds to the lower limit of protective mechanical ventilation, and has been shown to lead to low mechanical power (Samary et al., 2016b) in rats. The V_T_ sizes of 13 and 22 mL/kg were chosen because they have been shown to promote VILI when mechanical power is not kept within a safe limit (Samary et al., 2016b). Settings were maintained for 2 h. Respiratory system mechanics were measured at 5 min (INITIAL), 60 min, and at the end of the experiment (FINAL). At FINAL, arterial blood gases were analyzed, heparin (1000 IU) was injected into the tail vein. The trachea was then clamped at the same airway pressure at end-expiration, at PEEP = 3 cmH_2_O, and all animals were killed by overdose of sodium thiopental (60 mg/kg i.v.) followed by transection of the abdominal aorta and vena cava. Lungs were then extracted for histology and molecular biology analysis.

### Data acquisition and processing

A pneumotachograph (internal diameter = 1.5 mm, length = 4.2 cm, distance between side ports = 2.1 cm) was connected to the tracheal cannula for airflow (V′) measurements (Mortola and Noworaj, 1983). The pressure gradient across the pneumotachograph was determined using a SCIREQ differential pressure transducer (UT-PDP-02, SCIREQ, Montreal, QC, Canada). The flow resistance of the equipment, tracheal cannula included, was constant up to flow rates of 26 mL.s^−1^ and amounted to 0.12 cmH_2_O.mL^−1^.s ^−1^. The equipment dead space was 0.3 mL. Airflow, airway pressure (Paw, measured at the tip of the tracheal cannula), and Pes were continuously recorded throughout the experiments with a computer running custom-made software written in LabVIEW (National Instruments, Austin, TX) (Samary et al., 2015; Spieth et al., 2015). Briefly, V_T_ was calculated by digital integration of the airflow signal obtained from a custom-made pneumotachograph (Mortola and Noworaj, 1983) that was connected to the Y-piece of the ventilator tubing, while RR was calculated from the Pes swings as the frequency per minute of each type of breathing cycle.

Respiratory system and lung mechanics were calculated by occluding the airways at end-inspiration for 5 s until a respiratory system plateau pressure (Pplat,_RS_) and transpulmonary plateau pressure (Pplat,_L_), respectively, were reached (every 15 min) (Samary et al., 2015). Respiratory system driving pressure (ΔP,_RS_) was calculated as the difference between Pplat,_RS_ (post-end-inspiratory pause) and PEEP. Transpulmonary pressure (P,_L_) was calculated as the difference between the Paw and Pes, whereas transpulmonary driving pressure (ΔP,_L_) was the difference between the transpulmonary pressure during end-inspiration (post-inspiratory pause) and end-expiration. The mechanical energy (Energy,_L_) was calculated based on the equation described by Guerin et al. (2016) and Marini and Jaber (2016) (simplified formula), as Energy, _L_ = Δ${\text{P},}_{\text{L}}^{2}$/Est,_L_, where Est,_L_ is the static lung elastance. Energy, _L_ = Δ${\text{P},}_{\text{L}}^{2}$/(ΔP,_L_/V_T_) = ΔP,_L_xV_T_, which is the area of rectangle (see Supplementary Figure 1). Therefore, one must compute the area of the rectangle and divide the result by 2. Values were converted to mJ and multiplied by RR to obtain the mechanical power in mJ/min. This equation estimates the elastic work, the major component of the total work, without taking into account resistive properties and PEEP, unlike the equation proposed by Gattinoni et al. (2016). During the experiment, mechanical power was computed every 15 min to keep it at a low level. After finishing the experiments, all mechanical data were analyzed offline. At every 15 min of acquired data, a 3-to-5-min period of mechanical ventilation was assessed, and the values obtained during each of these periods were averaged for analysis.

All signals were amplified in a four-channel signal conditioner (SC-24, SCIREQ, Montreal, QC, Canada), and sampled at 200 Hz with a 12-bit analog-to-digital converter (National Instruments; Austin, Texas, USA). Mechanical data were computed offline by a routine written in MATLAB (Version R2007a; The Mathworks Inc., Natick, Massachusetts, USA).

### Histology

#### Light microscopy

The lungs and heart were removed *en bloc*. The left lung was frozen in liquid nitrogen and immersed in formaldehyde solution (4%), embedded in paraffin, cut longitudinally in the central zone by means of a microtome into three slices, each 4 μm thick, and stained with hematoxylin–eosin for histological analysis (Samary et al., 2015; Padilha et al., 2016). Photomicrographs at magnifications of ×100, ×200, and ×400 were obtained from eight nonoverlapping fields of view per section using a light microscope (Olympus BX51, Olympus Latin America Inc., Brazil). DAD was quantified using a weighted scoring system by two investigators (V.M. and V.L.C.) blinded to group assignment and independently, as described elsewhere (Kiss et al., 2016). Briefly, scores of 0–4 were used to represent edema, atelectasis, and overdistension, with 0 standing for no effect and 4 for maximum severity. Additionally, the extent of each scored characteristic per field of view was determined on a scale of 0–4, with 0 standing for no visible evidence and 4 for complete involvement. Scores were calculated as the product of severity and extent of each feature, on a range of 0–16. The cumulative DAD score was calculated as the sum of each score characteristic and ranged from 0 to 48.

#### Transmission electron microscopy

Three slices (2 × 2 × 2 mm) were cut from three different segments of the right lung and fixed in 2.5% glutaraldehyde in 0.1M Sorensen's Sodium-Potassium Phosphate Buffer. They were then stained *en bloc* in aqueous uranyl acetate for 24 h. After this procedure, samples were dehydrated in acetone and embedded in Araldite® resin. Ultrathin sections were obtained with a diamond knife on an LKB Ultratome microtome (Leica, Deerfield, IL, USA), placed on a 200 mesh copper grid (Lab Research Industries, Burlington, VT, USA), and counterstained with uranyl acetate and lead citrate. Micrographs were taken at 80 kV with a JEOL 100cx 100KW transmission electron microscope (Philips, Munich, Germany). For each lung electron microscopy image (20 fields per animal), type II epithelial cell damage, extracellular matrix (ECM) injury, and endothelial cell damage were graded on a five-point, semiquantitative, severity-based scoring system as follows: 0 = normal lung parenchyma, 1–4 = changes in 1–25%, 26–50%, 51–75%, and 76–100% of examined tissue, respectively (Samary et al., 2015). Electron microscopy analyses were performed by two investigators (V.M. and V.L.C.) blinded to group assignment.

#### Biological markers

Quantitative real-time reverse transcription polymerase chain reaction (PCR) was performed by an investigator (C.L.S.) blinded to group assignment in order to measure biomarkers associated with inflammation [interleukin (IL)-6], mechanical pulmonary stretch (amphiregulin), epithelial cell damage [club cell secretory protein 16 (CC16)], endothelial cell damage [intercellular adhesion molecular (ICAM)-1], extracellular matrix damage (syndecan-1 and decorin), and metalloproteinase (MMP)-9. The primers used are described in the Supplementary Table 1. Central slices of the right lung were cut, collected in cryotubes, flash-frozen by immersion in liquid nitrogen, and stored at −80 °C. Total RNA was extracted from frozen tissues using the RNeasy Plus Mini Kit (Qiagen, Hilden, Germany), following the manufacturer's recommendations. RNA concentrations were measured by spectrophotometry in a Nanodrop ND-1000 system (Thermo Scientific, Wilmington, DE, USA). First-strand cDNA was synthesized from total RNA using a Quantitec reverse transcription kit (Qiagen, Hilden, Germany). Relative mRNA levels were measured with a SYBR green detection system in an ABI 7500 real-time PCR system (Applied Biosystems, Foster City, California, USA). Samples were run in triplicate. For each sample, the expression of each gene was normalized to the acidic ribosomal phosphoprotein P0 (*36B4*) housekeeping gene (Akamine et al., 2007) and expressed as fold change relative to respective NV animals, using the 2^−ΔΔ*Ct*^ method, where ΔCt = Ct (target gene) – Ct (reference gene) (Schmittgen and Livak, 2008).

#### Statistical analysis

The sample size was calculated to allow detection of the lowest, but still significant, changes in IL-6 between ventilatory strategies, which was expected between V_T_ = 6 and 13 mL/kg and derived from a previous publication of our group (Samary et al., 2015). A sample size of 7 animals per group would provide the appropriate power (1 – β = 0.8) to identify significant differences in IL-6 (adjusted α = 0.025 for two comparisons), taking into account an effect size *d* = 2.0, a two-sided *t-*test, and a sample size ratio = 1 (G^*^Power 3.1.9.2, University of Düsseldorf, Düsseldorf, Germany).

Data were tested for normality using the Kolmogorov-Smirnov test with Lilliefors' correction, while the Levene median test was used to evaluate the homogeneity of variances. If both conditions were satisfied, time-dependent differences among groups were determined with general linear models followed by Bonferroni tests. Molecular biology, DAD, and electron microscopy variables were assessed with the Kruskal-Wallis test followed by Dunn's test. Multiple linear regression analyses between pHa, PaCO_2_, PaO_2_/FiO_2_, DAD score, IL-6, amphiregulin, CC16, decorin, syndecan-1, and MMP-9 (dependent variables, known as outcome variables) and V_T_, ΔP,_L_, and RR (independent variables, known as predictor variables), which represent the mathematical components of mechanical power, were performed. Parametric data were expressed as mean ± SD, while nonparametric data were expressed as median (interquartile range). General linear models and multiple linear regression analyses were carried out in SPSS 11.5.1 (SPSS Inc., Chicago, IL, USA). All the other tests were carried out in GraphPad Prism 6.00 (GraphPad Software, La Jolla, CA, USA). Significance was established at *p* < 0.05.

## Results

**Figure 2:**
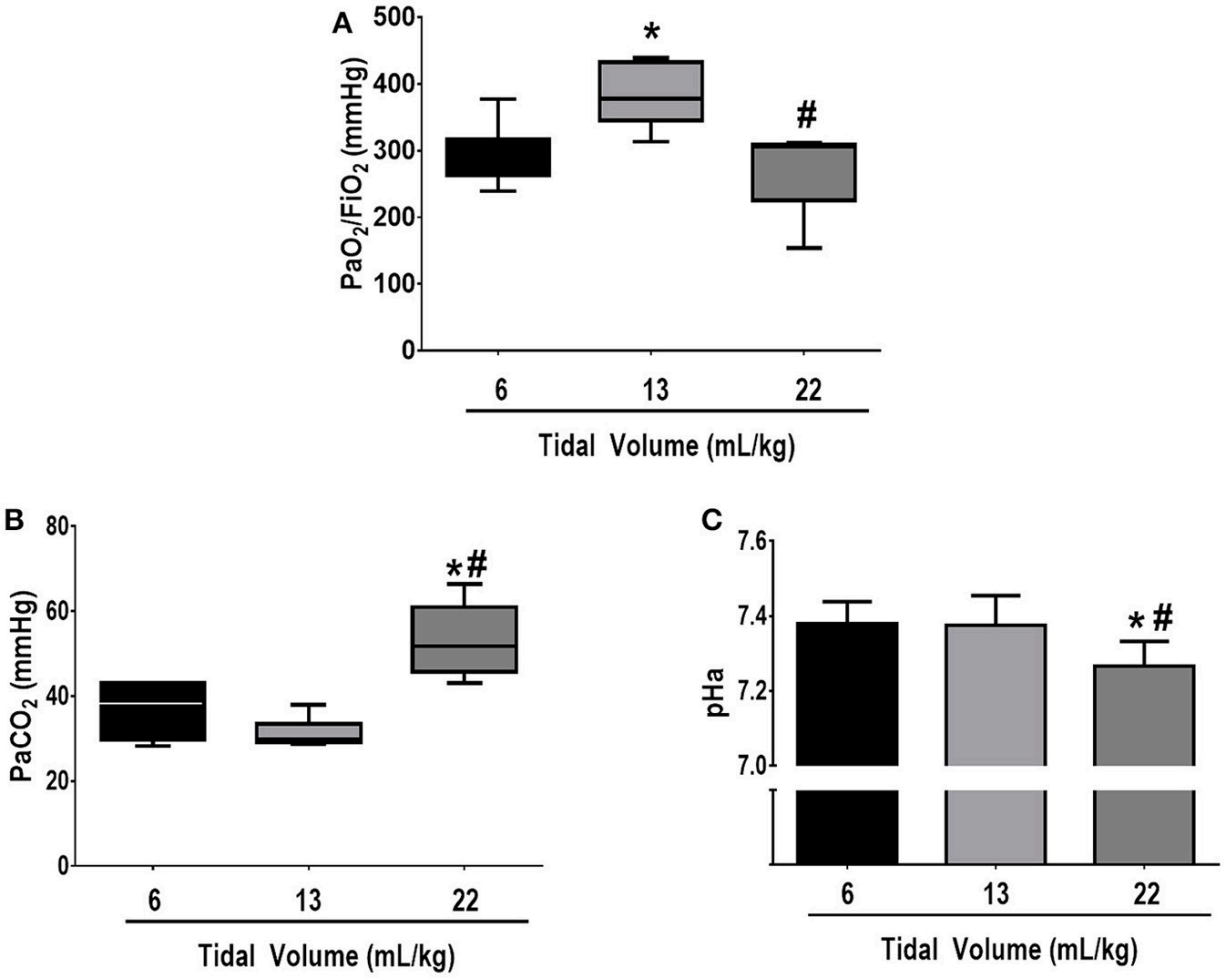
**(A)** PaO_2_/FiO_2_, **(B)** PaCO_2_, and **(C**) arterial pH at FINAL. The following groups were analyzed: (1) V_T_ = 6 mL/kg and RR adjusted to normocapnia; (2) V_T_ = 13 mL/kg; (3) V_T_ = 22 mL/kg. In the second and third groups, RR was adjusted to yield mechanical power comparable to that in the first group. PaO_2_/FiO_2_ and PaCO_2_ values are medians and interquartile ranges of 7 rats in each group. pH values are mean (SD) of 7 animals/group. *vs. 6 mL/kg (*p* < 0.05); ^#^vs. 13 mL/kg (*p* < 0.05).

Respiratory parameters and blood gas exchange at BASELINE were comparable among groups (see Supplementary Table 2). Mean arterial pressure was >70 mmHg throughout the experiments in all groups. At FINAL, no significant differences among groups were observed in the volume of fluids required to keep MAP >70 mmHg (V_T_ = 6 mL/kg: 12 ± 6 mL, V_T_ = 13 mL/kg: 12 ± 1 mL, V_T_ = 22 mL/kg: 14 ± 5 mL). Oxygenation was higher in V_T_ = 13 mL/kg compared to V_T_ = 6 mL/kg (*p* = 0.03) and V_T_ = 22 mL/kg (*p* = 0.02). PaCO_2_ was higher whereas pHa was lower in V_T_ = 22 mL/kg compared to V_T_ = 13 mL/kg and V_T_ = 6 mL/kg (*p* = 0.001 and *p* = 0.03, respectively) (Figure 2).

With the progressive increase in V_T_, RR reduced significantly in order to keep comparable mechanical power among groups (Table 1). Airflow reduced significantly in V_T_ = 22 mL/kg compared to V_T_ = 6 and V_T_ = 13 mL/kg. Pplat,_RS_, ΔP,_RS_, Pplat,_L_, ΔP,_L_ and Energy,_L_ were higher in V_T_ = 22 mL/kg in comparison to V_T_ = 6 and 13 mL/kg. Pplat,_RS_ and ΔP,_RS_ were higher in V_T_ = 13 compared to 6 mL/kg, while no differences were observed in Pplat,_L_, ΔP,_L_, or Energy,_L_ between them.

**Table 1:** Respiratory parameters during mechanical ventilation.

**Parameter**	**V_T_ (mL/kg)**	**INITIAL**	**60 min**	**FINAL**	**Time effect**	**Group effect**	**Time vs. group effect**
V_T_ (mL/kg)					*p* = 0.89	*p* < 0.001	*p* = 0.99
	6	5.8 ± 0.3	5.9 ± 0.3	5.9 ± 0.4			
	13	12.7 ± 0.4^*^	12.9 ± 0.2^*^	12.9 ± 0.1^*^			
	22	22.1 ± 2.7^*^^#^	21.9 ± 3.3^*^^#^	22.5 ± 3.4^*^^#^			
RR (bpm)					*p* = 0.73	*p* < 0.001	*p =* 0.99
	6	70.1 ± 0.4	71.2 ± 2.2	72.1 ± 3.4			
	13	35.3 ± 10.6^*^	35.3 ± 10.6^*^	35.8 ± 8.8^*^			
	22	11.8 ± 2.9^*^^#^	13.4 ± 3.2^*^^#^	13.4 ± 2.9^*^^#^			
Flow (mL/s)					*p =* 0.52	*p* < 0.001	*p =* 0.98
	6	11.8 ± 2.7	12.3 ± 2.7	12.9 ± 2.7			
	13	12.5 ± 2.7	12.5 ± 2.5	12.9 ± 1.6			
	22	5.9 ± 0.8^*^^#^	6.7 ± 0.8^*^^#^	6.8 ± 0.7^*^^#^			
Pplat,_RS_ (cmH_2_O)					*p =* 0.76	*p* < 0.001	*p =* 0.59
	6	11.1 ± 1.0	11.5 ± 0.9	12.3 ± 1.0			
	13	15.7 ± 2.9^*^	15.5 ± 2.3^*^	15.3 ± 1.5^*^			
	22	20.9 ± 3.4^*^^#^	19.6 ± 1.9^*^^#^	20.1 ± 1.6^*^^#^			
ΔP,_RS_ (cmH_2_O)					*p =* 0.76	*p* < 0.001	*p =* 0.54
	6	7.9 ± 1.0	8.5 ± 0.8	9.2 ± 0.9			
	13	12.6 ± 2.9^*^	12.4 ± 2.2^*^	12.4 ± 1.6^*^			
	22	17.8 ± 3.4^*^^#^	16.6 ± 1.9^*^^#^	17.1 ± 1.6^*^^#^			
Pplat,_L_ (cmH_2_O)					*p =* 0.76	*p* < 0.001	*p =* 0.59
	6	10.4 ± 1.3	10.6 ± 1.2	11.2 ± 0.8			
	13	13.3 ± 2.9^*^	13.1 ± 2.4^*^	12.8 ± 1.9			
	22	19.2 ± 3.3^*^^#^	17.8 ± 1.9^*^^#^	17.9 ± 2.2^*^^#^			
ΔP,_L_ (cmH_2_O)					*p =* 0.80	*p* < 0.001	*p =* 0.54
	6	7.2 ± 1.4	7.6 ± 1.2	8.2 ± 0.8			
	13	10.3 ± 3.0^*^	10.1 ± 2.4^*^	9.9 ± 2.0			
	22	16.2 ± 3.3^*^^#^	14.8 ± 1.9^*^^#^	14.9 ± 2.2^*^^#^			
Energy,L (mJ)					*p =* 0.78	*p* < 0.001	*p =* 0.79
	6	0.93 ± 0.16	1.01 ± 0.16	1.11 ± 0.11			
	13	2.28 ± 0.98^*^	2.27 ± 0.94^*^	2.20 ± 0.80^*^			
	22	6.11 ± 2.13^*^^#^	5.48 ± 1.32^*^^#^	5.48 ± 0.84^*^^#^			
Power (mJ/min)					*p =* 0.06	*p =* 0.48	*p =* 0.38
	6	65 ± 11	71 ± 11	78 ± 8			
	13	73 ± 8	72 ± 7	73 ± 5			
	22	68 ± 7	70 ± 4	72 ± 11			

Respiratory parameters during mechanical ventilation in the following groups: (1) V_T_ = 6 mL/kg and RR adjusted to normocapnia; (2) V_T_ = 13 mL/kg; (3) V_T_ = 22 mL/kg. In the second and third groups, RR was adjusted to yield mechanical power comparable to that in the first group. Values are mean ± standard deviation (SD) of 8 animals/group. Comparisons were evaluated among time, groups, and time vs. group and according to general linear models followed by pairwise comparisons (p < 0.05).

* vs. V_T_ = 6 mL/kg;

# *vs. V_T_ = 13 mL/kg*.

**Figure 3:**
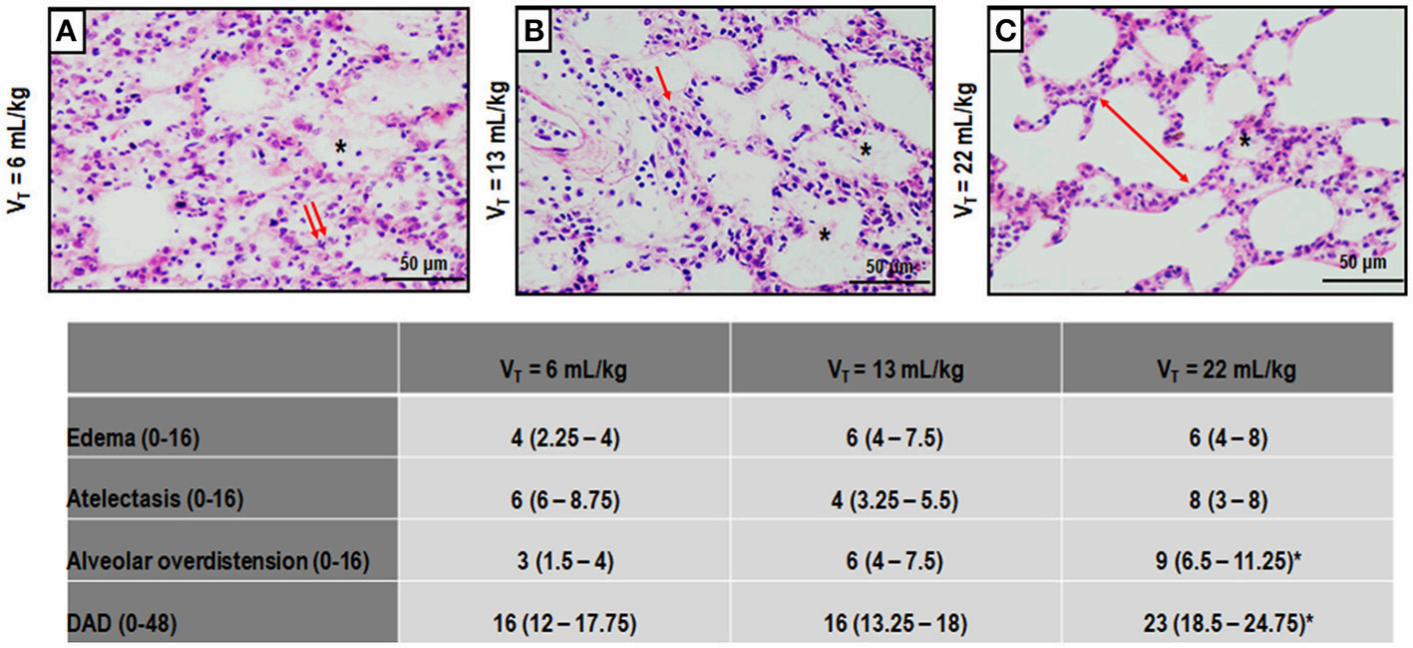
Photomicrographs (light microscopy) of lung parenchyma stained with hematoxylin and eosin. The following groups were analyzed: (1) V_T_ = 6 mL/kg and RR adjusted to normocapnia **(A)**; (2) V_T_ = 13 mL/kg **(B)**; (3) V_T_ = 22 mL/kg **(C)**. In the second and third groups, RR was adjusted to yield mechanical power comparable to that in the first group. Asterisks show alveolar/interstitial edema; arrows indicate alveolar collapse, and double arrows, areas of alveolar overdistension. Photomicrographs are representative of data obtained from lung sections of seven animals (original magnification, 200×). Bars = 100 μm. Cumulative diffuse alveolar damage (DAD) score representing injury from alveolar/interstitial edema, atelectasis, and alveolar overdistension. Values are median (interquartile range) of 7 animals/group. *vs. V_T_ = 6 mL/kg (*p* < 0.05).

**Figure 4:**
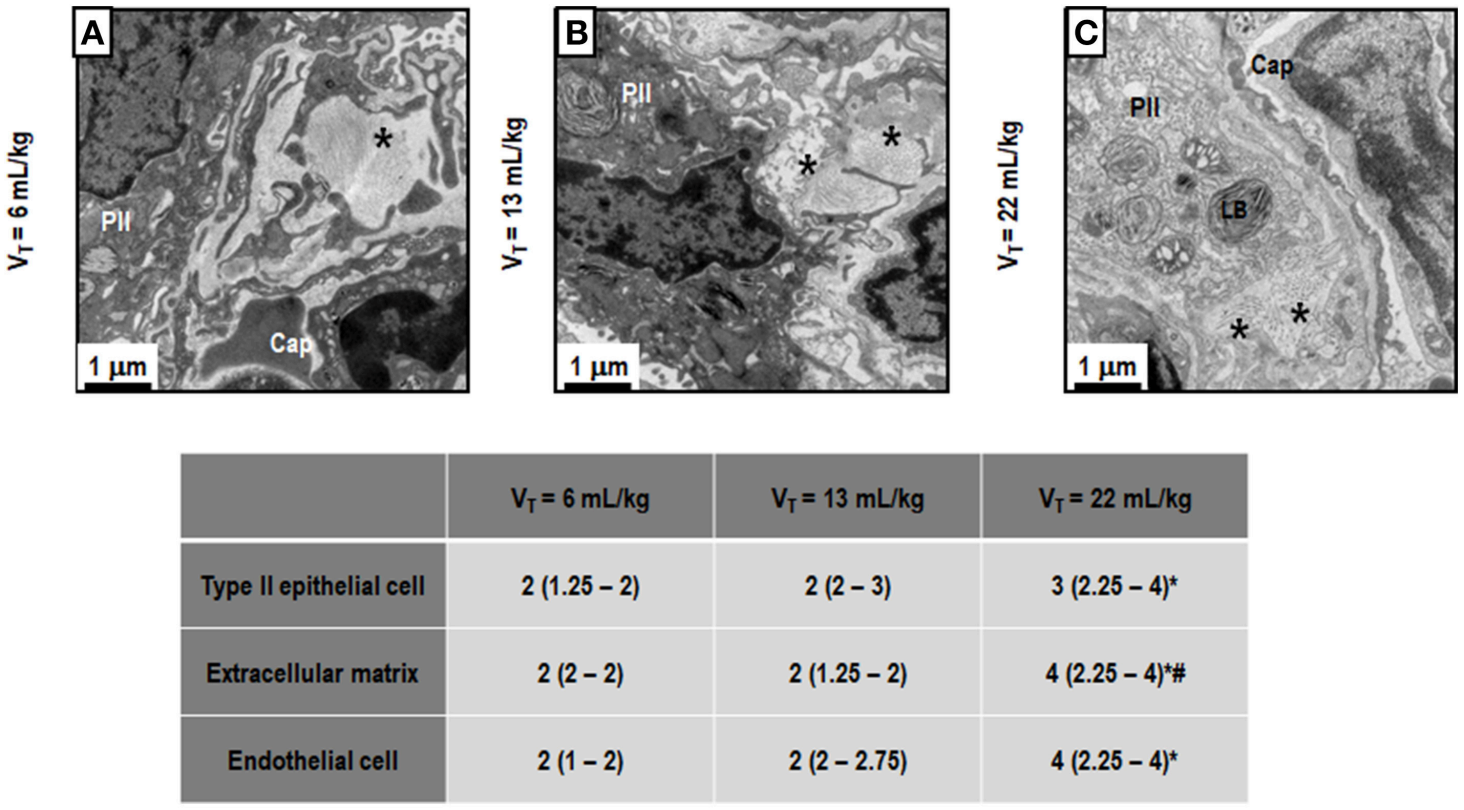
Transmission electron microscopy of alveolar architecture. Photomicrographs are representative of data obtained from lung sections of 7 animals in each group. The following groups were analyzed: (1) V_T_ = 6 mL/kg and RR adjusted to normocapnia **(A)**; (2) V_T_ = 13 mL/kg **(B)**; (3) V_T_ = 22 mL/kg **(C)**. In the second and third groups, RR was adjusted to yield mechanical power comparable to that in the first group. Asterisk: extracellular matrix (ECM), PII: Type II epithelial cell, Cap: capillary, LB, lamellar body. PII damage is visible in all ARDS groups, regardless of tidal volume. LB decreased with increasing tidal volume. Moreover, the ECM was characterized by damage to collagen/elastic fibers (asterisk). Endothelial cells were less damaged at V_T_ = 6 mL/kg and V_T_ = 13 mL/kg. Semi-quantitative electron microscopy analysis of lung tissue. A five-point, semi-quantitative, severity-based scoring system was used: 0 = normal; or damage to 1 = 1–25%; 2 = 26–50%; 3 = 51–75%; and 4 = 76–100% of examined tissue. Values are median (interquartile range) of 7 animals/group. *vs. V_T_ = 6 mL/kg (*p* < 0.05); ^#^vs. V_T_ = 13 mL/kg (*p* < 0.05).

Figure 3 depicts light microscopy images of representative animals. V_T_ = 22 mL/kg resulted in a higher DAD score compared to V_T_ = 6 mL/kg, mainly due to overdistension. Electron microscopy images of one representative animal per group are shown in Figure 4. V_T_ = 22 mL/kg increased damage to type II epithelial cells and endothelial cells compared to V_T_ = 6 mL/kg. V_T_ = 22 mL/kg also resulted in greater injury to extracellular matrix (ECM) than V_T_ = 6 mL/kg and V_T_ = 13 mL/kg. Other transmission electron microscopy images detailing damage to type II epithelial cells, ECM, and endothelial cells are depicted in Supplementary Figure 2.

**Figure 5:**
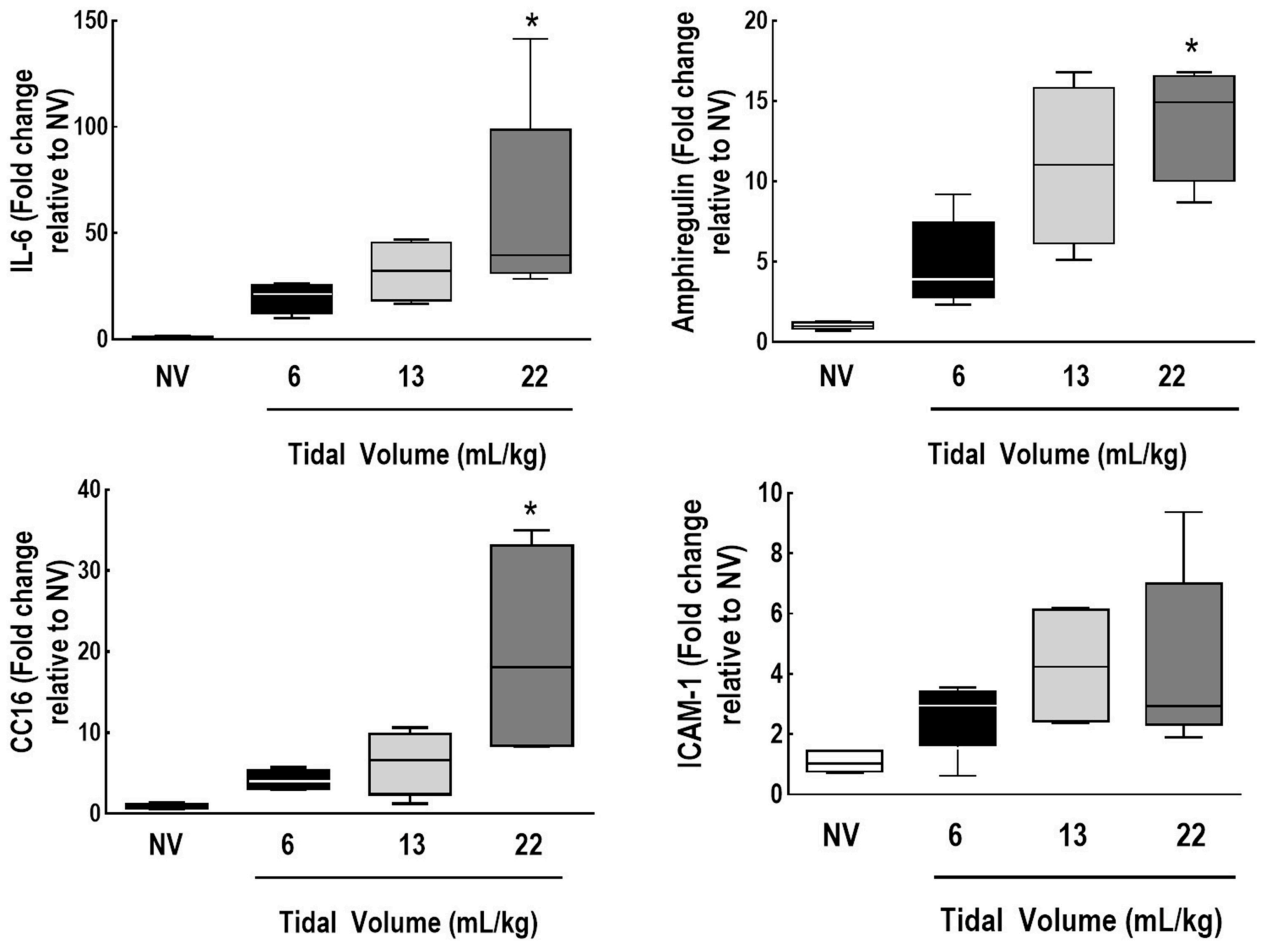
Expression of biomarkers associated with inflammation, alveolar stretch, and epithelial and endothelial cell damage. Real-time polymerase chain reaction analysis of markers of inflammation [interleukin (IL)-6], alveolar stretch (amphiregulin), and epithelial [club cell protein (CC)16] and endothelial cell damage [intercellular adhesion molecule (ICAM)-1]. Relative gene expression was calculated as the ratio of average gene expression levels to reference gene (*36B4*) expression and presented as fold change relative to non-ventilated animals (NV). Values are medians and interquartile ranges of 7 rats in each group. Comparisons among all groups were done by the Kruskal-Wallis test followed by Dunn's test (*p* < 0.05). *vs. V_T_ = 6 mL/kg (*p* < 0.05).

**Figure 6:**
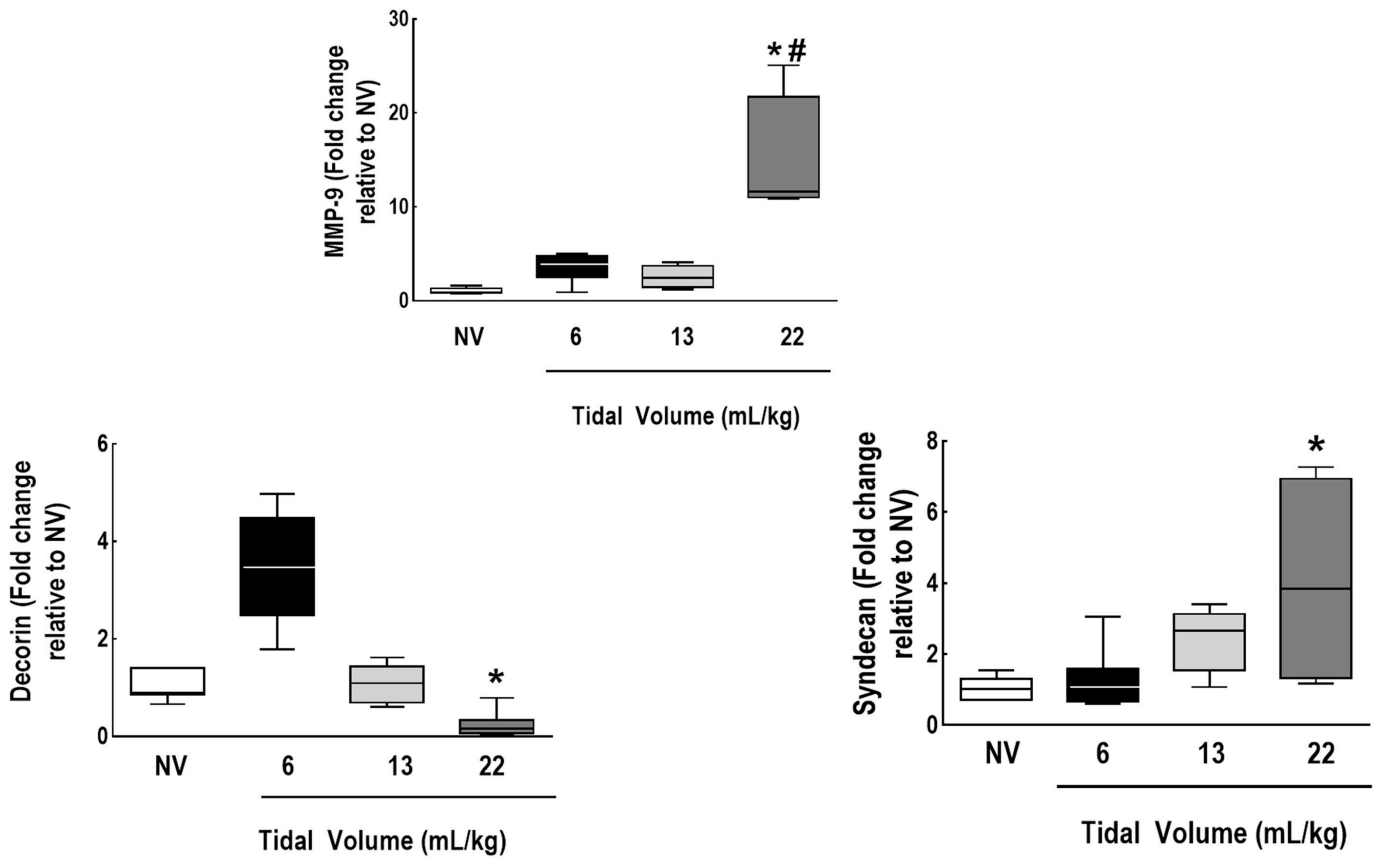
Expression of biomarkers associated with extracellular matrix damage and metalloproteinase. Real-time polymerase chain reaction analysis of markers of extracellular matrix damage (syndecan-1 and decorin) and of metalloproteinase (MMP)-9. Relative gene expression was calculated as the ratio of average gene expression levels to reference gene (*36B4*) expression and presented as fold change relative to non-ventilated animals (NV). Values are medians and interquartile ranges of 7 rats in each group. Comparisons among all groups were done by the Kruskal-Wallis test followed by Dunn's test (*p* < 0.05). *vs. V_T_ = 6 mL/kg (*p* < 0.05). ^#^vs. V_T_ = 13 mL/kg (*p* < 0.05).

IL-6, amphiregulin, and CC16 expressions were higher in V_T_ = 22 mL/kg compared to V_T_ = 6 mL/kg (Figure 5). Syndecan expression was higher in V_T_ = 22 mL/kg, while decorin expression was lower, in comparison to V_T_ = 6 mL/kg. MMP-9 was higher in V_T_ = 22 mL/kg than V_T_ = 6 mL/kg and 13 mL/kg (Figure 6).

On multiple linear regression analyses, V_T_ best predicted changes in IL-6 and CC16, while ΔP,_L_ best predicted variance in pHa, PaO_2_/FiO_2_, and amphiregulin (Table 2). Syndecan was predicted by V_T_ and ΔP,_L_. RR only predicted changes in decorin. The *r*^2^-values from multiple linear regression, when components of Energy,_L_ (V_T_, ΔP,_L_, and RR) were used, were compared to *r*^2^-values from the linear regression performed using Energy,_L_ which encompasses all of these components. Interestingly, the isolated components of Energy,_L_, mainly V_T_, predicted changes in IL-6 better than the Energy,_L_ construct did (*r*^2^ = 0.71 vs. 0.19, respectively), while DAD was comparable (*r*^2^ = 0.46 vs. 0.47) (Table 2; see Supplementary Table 3).

**Table 2:** Coefficients of multiple linear regression.

	**V_T_**	**ΔP,_L_**	**RR**	***r*^2^**
pHa	0.002 (–0.009, 0.014)	–0.22 (–0.04, –0.006)^*^	0.001 (–0.003, 0.003)	0.57
PaCO_2_ (mmHg)	0.44 (−1.22, 2.11)	1.82 (–0.63, 4.26)	0.07 (–0.42, 0.56)	0.43
PaO_2_/FiO_2_	–4.03 (–14.35, 6.29)	–22.72 (–37.74, –7.70)^*^	–3.56 (–6.54, –0.57)	0.46
DAD score	0.07 (–0.67, 0.82)	1.08 (–0.18, 2.18)	0.03 (–0.19, 0.25)	0.46
IL-6 (fold change relative to NV)	7.1 (2.7, 11.4)^*^	–3.4 (–14.9, 8.1)	0.9 (–1.1, 2.9)	0.71
Amphiregulin (fold change relative to NV)	0.6 (–0.3, 1.3)	–1.8 (–3.3, –0.3)^*^	–0.1 (–0.4, 0.1)	0.68
CC 16 (fold change relative to NV)	1.7 (0.2, 3.2)^*^	0.7 (–2.9, 4.4)	0.3 (–0.3, 0.9)	0.68
Decorin (fold change relative to NV)	0.1 (–0.1, 0.1)	0.2 (–0.03, 0.4)	0.1 (0.03, 0.1)^*^	0.86
Syndecan (fold change relative to NV)	0.4 (0.1, 0.6)^*^	–0.5 (–0.9, –0.03)^*^	0.001 (–0.1, 0.1)	0.65
MMP-9 (fold change relative to NV)	0.5 (–0.5, 1.6)	1.26 (–1.2, 3.7)	0.16 (–0.23, 0.54)	0.39

Multiple linear regression analyses between pHa, PaCO2, PaO_2_/FiO_2_, DAD score, IL-6, amphiregulin, CC16, decorin, syndecan-1, and MMP-9 (dependent variables) and V_T_, ΔP,_L_, and RR (independent variables), which represent the mathematical components of mechanical power. Values represent the β coefficients (95%CI) of multiple linear regression analyses. r^2^ represents the percentage of the response variable variation that is explained by the multiple linear model.

* *p < 0.05*.

## Discussion

In the rat model of mild-to-moderate ARDS used herein, we found that, at low mechanical power, higher V_T_ resulted in increased PaCO_2_, DAD scores, and gene expression of mediators associated with inflammation (IL-6), alveolar stretch (amphiregulin), damage to epithelial cells (CC16), and extracellular matrix (MMP-9 and syndecan-1). Furthermore, only V_T_ was able to predict changes in IL-6 and CC16, whereas ΔP,_L_ predicted changes in pHa, oxygenation, amphiregulin, and syndecan-1 expression. Thus, even when mechanical power was low, high V_T_ resulted in VILI. Changes in PaCO_2_ might be considered as a parameter to monitor for VILI development even at low mechanical power. Our findings suggest that V_T_ is a better determinant of lung injury than mechanical power or RR.

To the best of our knowledge, this was the first study to investigate the impact of different V_T_ levels, under the same low mechanical power, on lung function, DAD score, ultrastructural analysis of pulmonary parenchyma and gene expression of different biomarkers of VILI in experimental ARDS. We calculated the mechanical power by using a simplified equation to allow possible extrapolation of results to the clinical scenario (Marini and Jaber, 2016). PEEP was kept constant throughout the experiment; thus, different V_T_ levels led only to dynamic energy changes. A particular strength of our study was the tight control of low mechanical power and the prospective nature of the investigation. Low mechanical power was ensured by ventilating animals with low V_T_ levels (protective mechanical ventilation) and setting the RR to keep gas exchange and pHa within physiologic limits. Thus, minute ventilation was kept through the modulation of V_T_ and RR. Lung damage was evaluated by light and electron microscopy and partitioned into its components, such as epithelial, endothelial, and extracellular matrix injury. Additionally, the severity of lung injury (mild to moderate) induced in this model corresponds to that observed in approximately two-thirds of ARDS cases (Bellani et al., 2016).

Recently, it was suggested that the various potential causes of VILI might be unified in a single variable, such as mechanical power. This variable is measured by the pressure–volume loop and can be computed from its components, such as V_T_, ΔP,_L_, airflow, PEEP, and RR. Thus, the relative contributions of each of these component variables to mechanical power as a determinant of VILI can be evaluated. Previous theoretical (Gattinoni et al., 2016) and experimental (Cressoni et al., 2016) studies suggested that lung damage is the result of the energy and power delivered to the lungs. The quantification of mechanical power at bedside and its interpretation might be helpful to guide optimization of settings for “safe” mechanical ventilation. The main hypothesis of the present study was that VILI at low mechanical power would be independent of delivered V_T_. In fact, low mechanical power may be achieved by setting lower V_T_ and higher RR or vice-versa.

A previous theoretical study (Gattinoni et al., 2016) hypothesized that the main determinants of energy and power would be V_T_, ΔP,_L_, and airflow, followed by RR. Our findings suggest that, at low mechanical power, higher V_T_ plays a greater role than RR as a determinant VILI, yielding higher respiratory system and lung Pplat and ΔP. RR has been recognized as an early potential factor underlying lung injury (Hotchkiss et al., 2000), mainly when associated with increased power (Cressoni et al., 2016).

Increased DAD scores and damage to the epithelium, extracellular matrix, and endothelial cells were observed only at V_T_ = 22 mL/kg. Therefore, at low mechanical power, there was probably a threshold of inspiratory stress and dynamic strain after which the lung epithelium, endothelium, and ECM started to sustain further injury. Lung morphology changes were followed by increased expression of IL-6, a pro-inflammatory mediator, and biomarkers of epithelial and extracellular matrix damage. This suggests that the proportional role of V_T_ in promoting lung damage is greater than predicted by the mechanical power model, which, in turn, is likely explained by the fact that high V_T_ and the transpulmonary driving pressure used herein may have exceeded the safe limits of lung elasticity (Protti et al., 2011; Cressoni et al., 2015).

A previous study investigated the role of mechanical power in VILI in healthy piglets (Cressoni et al., 2016). Two experiments were attempted: V_T_ was kept constant and RR increased progressively; and V_T_ was increased while keeping the RR constant. Lung damage was evaluated through the increase in lung weight, assessed by CT scanning. They showed that lung edema increased while oxygenation decreased proportional to RR. We are unable to compare our results with those reported (Cressoni et al., 2016), due to major methodological differences: in the present study, lung damage was induced by endotoxin, mechanical power was kept low and constant while different combinations of V_T_ and RR were trialed, and VILI was evaluated not by lung weight but by histological analysis under light and electron microscopy, as well as by gene expression of biomarkers associated with inflammation, alveolar stretch, epithelial and ECM damage. To date, no other experimental studies have assessed the impact of power on VILI in ARDS.

The damage caused by higher V_T_ is not limited to epithelial and endothelial cells; it also affects ECM components. In this line, higher stress and strain may cause displacement or rupture of the ECM (Moriondo et al., 2007; Pelosi and Negrini, 2008; Protti et al., 2015). In the presence of high V_T_ (high dynamic strain), MMP-9 activity has been shown to increase significantly (Gonzalez-Lopez et al., 2011; Jain et al., 2017). Accordingly, in our study, MMP-9 expression was higher in V_T_ = 22 mL/kg compared to V_T_ = 13 mL/kg and V_T_ = 6 mL/kg. MMP-9 cleaves collagen, but also increases neutrophil influx, contributing to the ongoing inflammatory process (Gaggar and Weathington, 2016). Gene expression of syndecan-1, a cell surface heparan sulfate proteoglycan primarily expressed in the lung epithelium and involved in regulation of lung repair, remodeling (Brauer et al., 2016), and inflammation (Hayashida et al., 2009), was higher in V_T_ = 22 mL/kg compared to V_T_ = 6 mL/kg. Since the lung epithelium is damaged by high V_T_ and syndecan-1 is expressed to regulate lung inflammation, we may postulate that syndecan-1 may also contribute to lung inflammation in VILI. Both V_T_ and ΔP,_L_ predicted changes in syndecan-1 expression, highlighting the sensitivity of this biomarker to detect changes in ECM homeostasis. In line with this result, decorin, which has been associated with inhibition of cell proliferation (Iacob et al., 2008) and collagen synthesis (Jahanyar et al., 2007), was lower in V_T_ = 22 mL/kg compared to V_T_ = 6 mL/kg. These findings suggest that, at low mechanical power, high V_T_ increased ECM fragmentation (Moriondo et al., 2007; Pelosi and Negrini, 2008), which may promote lung inflammation. In this context, gene expression of IL-6, and early surrogate of inflammation and VILI, was higher in the presence of higher V_T_. The higher gene expression of amphiregulin at V_T_ = 22 mL/kg might be attributed to overdistension (Dolinay et al., 2006). CC16, a protein mainly produced and secreted by club cells in the distal respiratory or terminal bronchioles, has been proposed as a biomarker of lung epithelial injury (Broeckaert et al., 2000) and its gene expression was also increased in V_T_ = 22 mL/kg. According to multiple linear regression analyses, changes in V_T_ better predicted changes in IL-6 and CC16 mRNA expressions. This can be explained by the importance of V_T_ in promoting lung injury under the same low mechanical power. Changes in ΔP,_L_ better reflected changes in amphiregulin mRNA expression, which has been observed in cell culture under cyclic deformations (Tschumperlin et al., 2000).

Even at low mechanical power, PaCO_2_ and end-expiratory alveolar overdistension also increased at higher V_T_. Since overall minute ventilation was comparable among groups, we hypothesize that alveolar dead space probably increased with lung injury. Previous studies showed that increased PaCO_2_ and dead space correlated with mortality (Nuckton et al., 2002), remodeling of lung parenchyma in ARDS (Gattinoni et al., 1993), and alveolar septal retraction due to high surface tension (Wilson, 1982). This suggests that monitoring changes in PaCO_2_ and dead space might be helpful to optimize protective mechanical ventilation settings, even at low mechanical power.

### Possible clinical implications

Our data reinforce the concept that mechanical power *per se* might not be a useful parameter to guide optimization protective mechanical ventilation. To minimize VILI, V_T_ seems to be the main ventilator parameter which should be controlled, irrespective of RR. In fact, the hypothesis that low mechanical power might be a major target to minimize VILI could erroneously result in acceptance of higher V_T_. Thus, even during mechanical ventilation at low mechanical power, inappropriately high V_T_ can still promote lung injury. We thus insist that, even when using low RR and ΔP,_L_, V_T_ should always be kept in low protective range. Certainly, this claim remains to be confirmed in clinical observational studies and randomized controlled trials.

### Limitations

This study has some limitations that should be considered. First, experimental ARDS was induced by endotoxin, and our results cannot be extrapolated to other models or to human ARDS. Second, as the observation time was only 2 h, we did not assess protein levels of biological markers. However, this duration of mechanical ventilation was sufficient to identify gene expression of markers of interest (Samary et al., 2015). Third, a simplified equation was chosen in order to facilitate its routine use in the clinical setting. This equation estimates the major component of the total work that is elastic work, without taking into account resistive properties and PEEP.

## Conclusion

In the experimental model of mild-to-moderate ARDS used herein, at low mechanical power, high V_T_ was associated with VILI. To mitigate VILI, V_T_ control seems more important than RR control.

## Author contributions

LM and PS participated in the design of the study, carried out the experiments, performed data analyses, and drafted the manuscript; AT, CS RS contributed to the study design and carried out the experiments; MF, VM, and VC carried out the experiments and lung morphology; LM performed analyses of lung mechanics; CS and MM carried out the molecular biology analyses and contributed to the manuscript; PP and MdA contributed to the study design, supervised the entire project, and helped write the manuscript; PR and PS contributed to the study design, supervised the experimental work and statistical analysis, wrote the manuscript, and supervised the entire project. All authors read and approved the final manuscript.

### Conflict of interest statement

The authors declare that the research was conducted in the absence of any commercial or financial relationships that could be construed as a potential conflict of interest.
