# Supplementary material for: Impact of Different Tidal Volume Levels at Low Mechanical Power on Ventilator-Induced Lung Injury in Rats
Source: Front Physiol. 2018 Apr 4;9:318. doi: 10.3389/fphys.2018.00318 (PMC5893648; doi:10.3389/fphys.2018.00318)
Supplement: Supplementary file 1 [file Image1.PDF]

Supplementary Material

Impact of different tidal volume levels at low mechanical power on ventilator-induced lung injury in rats

Lillian Moraes, Pedro L. Silva, Alessandra Thompson, Cintia L. Santos, Raquel S. Santos, Marcos V.S. Fernandes, Marcelo M. Morales, Vanessa Martins, Vera L. Capelozzi, Marcelo Gama de Abreu, Paolo Pelosi, Patricia R. M. Rocco*

*** Corresponding Author:** prmrocco@gmail.com

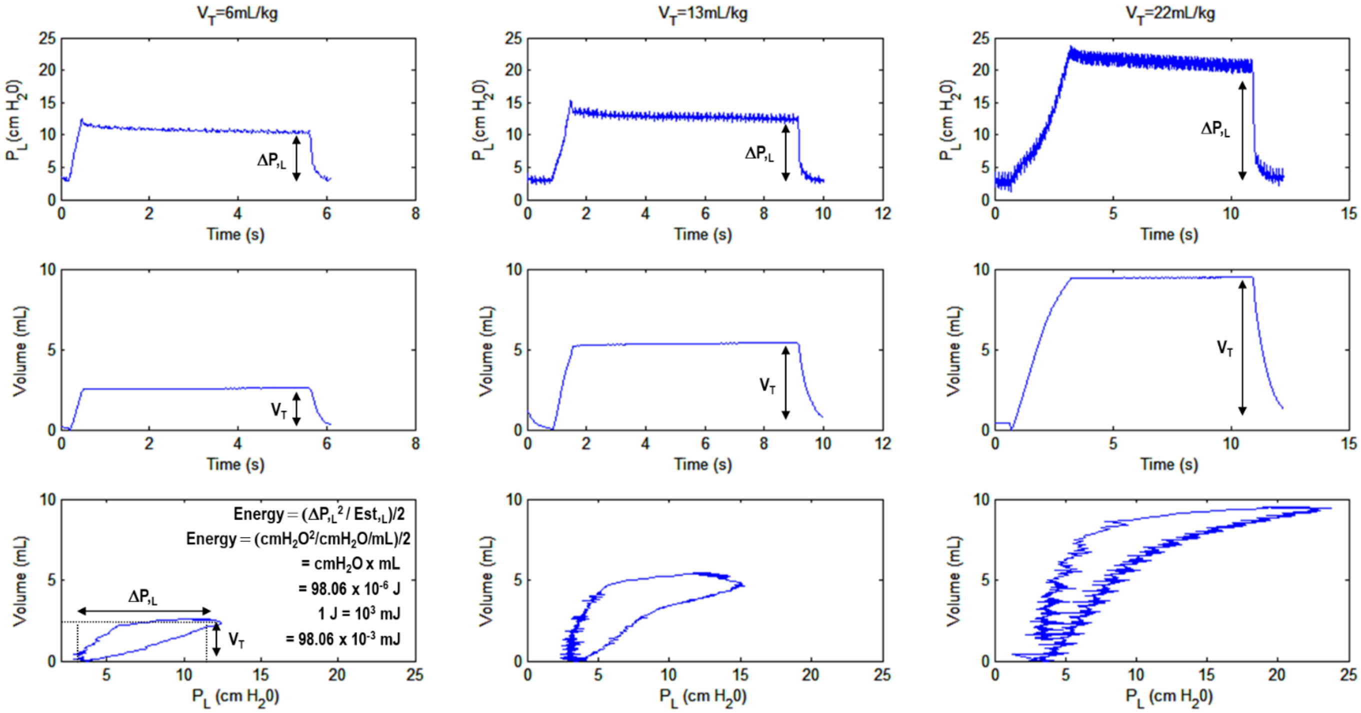

Supplementary Figure 1. Representative curves of transpulmonary pressure ($P_{,L}$), tidal volume, and volume-P,L curve. V_T: tidal volume, $\Delta P_{,L}$: transpulmonary driving pressure, Est,L: static lung elastance. The mechanical energy (Energy,L) was calculated based on the equation described by Guerin et al. (Guerin et al., 2016) and Marini and Jaber (Marini and Jaber, 2016) (simplified formula), as: $Energy_{,L} = \Delta P_{,L}{}^2/Est_{,L}. = \Delta P_{,L}{}^2/(\Delta P_{,L}/V_T) = \Delta P_{,L} x V_T$, which is the area of the rectangle. Therefore, one must compute the area of the rectangle and divide the result by 2. This simplified equation estimates elastic work without taking into account resistive properties and PEEP. Values were converted to mJ and multiplied by RR to obtain the mechanical power.
