# Supplementary material for: Impact of Different Tidal Volume Levels at Low Mechanical Power on Ventilator-Induced Lung Injury in Rats
Source: Front Physiol. 2018 Apr 4;9:318. doi: 10.3389/fphys.2018.00318 (PMC5893648; doi:10.3389/fphys.2018.00318)
Supplement: Supplementary file 2 [file Image2.PDF]

Supplementary Material

Impact of different tidal volume levels at low mechanical power on ventilator-induced lung injury in rats

Lillian Moraes, Pedro L. Silva, Alessandra Thompson, Cintia L. Santos, Raquel S. Santos, Marcos V.S. Fernandes, Marcelo M. Morales, Vanessa Martins, Vera L. Capelozzi, Marcelo Gama de Abreu, Paolo Pelosi, Patricia R. M. Rocco*

*** Corresponding Author:** prmrocco@gmail.com

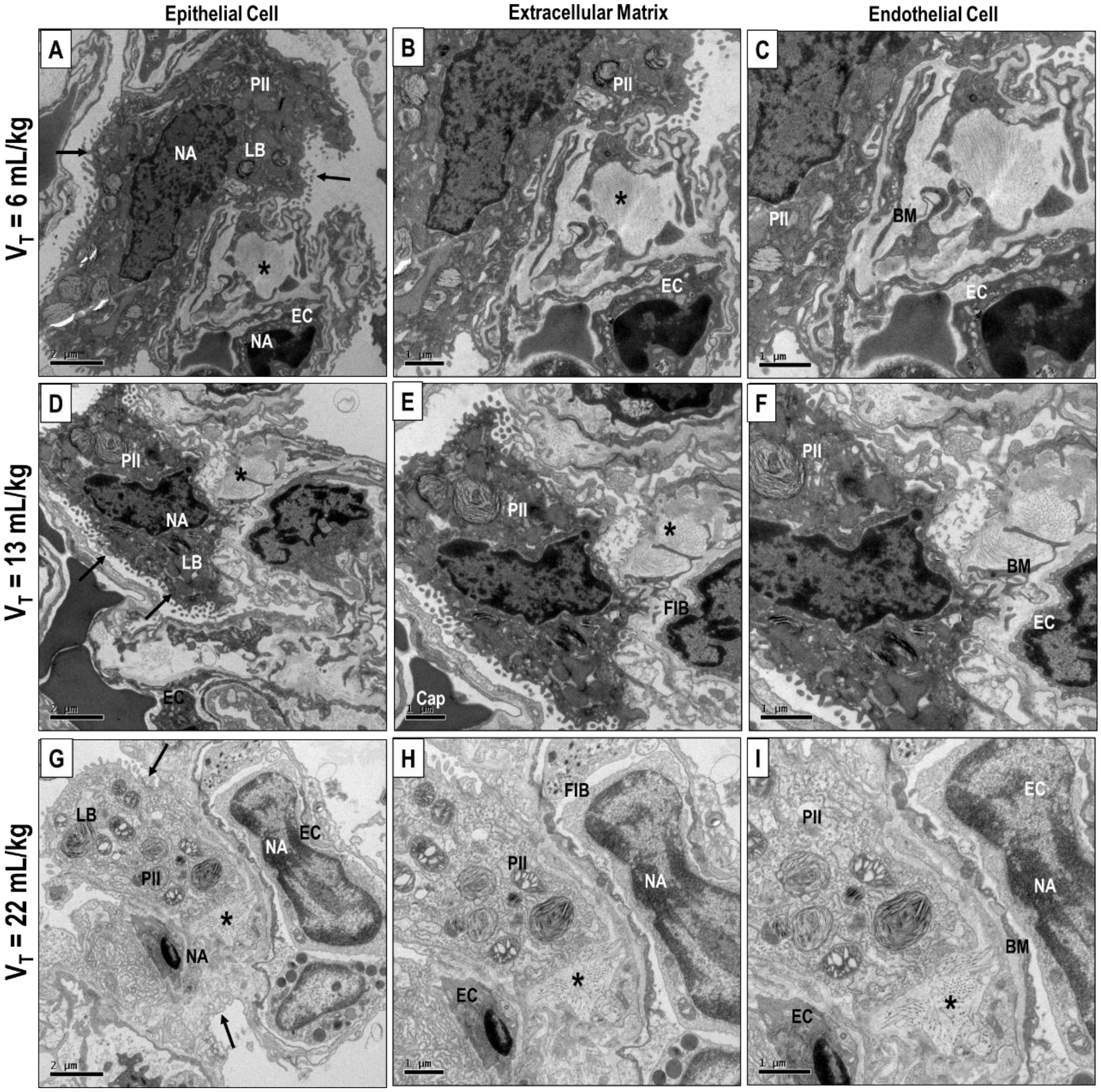

Supplementary Figure 2. Transmission electron microscopy of alveolar architecture. Photomicrographs are representative of data obtained from lung sections of 7 animals in each group. The following groups were analyzed: 1) V_T = 6 mL/kg and RR adjusted to normocapnia (A); 2) V_T = 13 mL/kg (B); 3) V_T = 22 mL/kg (C). In the second and third groups, RR was adjusted to yield mechanical power comparable to that in the first group. A, D, G: Note the progressive damage of type II epithelial cell (PII) characterized by nucleus apoptosis (NA), irregularity of cytoplasmic membrane (arrows) and decrease of lamellar bodies (LB). B,E,H: Note the increased number of fibroblasts (FIB) with the presence of types I and III collagen fibers (*). C, F, I: Note endothelial cell damage with apoptosis mainly in panel I.
