# Supplementary material for: Impact of Different Tidal Volume Levels at Low Mechanical Power on Ventilator-Induced Lung Injury in Rats
Source: Front Physiol. 2018 Apr 4;9:318. doi: 10.3389/fphys.2018.00318 (PMC5893648; doi:10.3389/fphys.2018.00318)
Supplement: Supplementary file 3 [file Table1.PDF]

Supplementary Material

Impact of different tidal volume levels at low mechanical power on ventilator-induced lung injury in rats

Lillian Moraes, Pedro L. Silva, Alessandra Thompson, Cintia L. Santos, Raquel S. Santos, Marcos V.S. Fernandes, Marcelo M. Morales, Vanessa Martins, Vera L. Capelozzi, Marcelo Gama de Abreu, Paolo Pelosi, Patricia R. M. Rocco*

*** Corresponding Author:** prmrocco@gmail.com

Supplementary Table 1. Forward and reverse oligonucleotide sequences of target gene primers used for analysis of biological markers associated with inflammation, alveolar stretch, epithelial and endothelial cell damage, and extracellular matrix injury.

**Gene**	**Primer**	**Primer sequences (5′-3′)**
IL-6	Forward	CTC CGC AAG AGA CTT CCA G
	Reverse	CTC CTC TCC GGA CTT GTG A
Amphiregulin	Forward	TTT CGC TGG CGC TCT CA
	Reverse	TTC CAA CCC AGC TGC ATA ATG
CC16	Forward	GAT CG CCA TCA CAA TCA CTG
	Reverse	GGT ATC CAC CAG CCT CTT CA
ICAM-1	Forward	CTT CCG ACT AGG GTC CTG AA
	Reverse	CTT CAG AGG CAG GAA ACA GG
Syndecan	Forward	GTT CCG CTG GTT TGT TGT TT
	Reverse	GAT GAA GGC TGT CCC AGG TA
Decorin	Forward	GAT CAG CCC AGA GGC ATT TA
	Reverse	GCT CCA TTT TCA ATC CCA GA
MMP-9	Forward	CCA CCG AGC TAT CCA CTC AT
	Reverse	GTC CGG TTT CAG CAT GTT TT
36B4	Forward	AAT CCT GAG CGA TGT GCA G
	Reverse	GCT GCC ATT GTC AAA CAC

IL, interleukin; CC, club cell protein; ICAM, intercellular adhesion molecule; MMP, metalloproteinase.
