# Supplementary material for: Impact of Different Tidal Volume Levels at Low Mechanical Power on Ventilator-Induced Lung Injury in Rats
Source: Front Physiol. 2018 Apr 4;9:318. doi: 10.3389/fphys.2018.00318 (PMC5893648; doi:10.3389/fphys.2018.00318)
Supplement: Supplementary file 4 [file Table2.PDF]

Supplementary Material

Impact of different tidal volume levels at low mechanical power on ventilator-induced lung injury in rats

Lillian Moraes, Pedro L. Silva, Alessandra Thompson, Cintia L. Santos, Raquel S. Santos, Marcos V.S. Fernandes, Marcelo M. Morales, Vanessa Martins, Vera L. Capelozzi, Marcelo Gama de Abreu, Paolo Pelosi, Patricia R. M. Rocco*

*** Corresponding Author:** prmrocco@gmail.com

Supplementary Table 2. Respiratory parameters and blood gas exchange at BASELINE

	V_T (mL/kg)		
	6	13	22
V_T (mL/kg)	5.8 ± 0.3	6.2 ± 0.3	6.4 ± 0.9
RR (bpm)	70.1 ± 0.3	70.1 ± 0.4	70.1 ± 0.4
Flow (mL/s)	11.8 ± 2.4	11.7 ± 1.9	9.7 ± 1.1
$Pplat,_{RS}$ (cmH_2O)	11.2 ± 1.4	10.4 ± 3.1	11.2 ± 1.6
$\Delta P,_{RS}$ (cmH_2O)	8.1 ± 1.4	7.4 ± 3.0	8.2 ± 1.6
$Pplat,_{L}$ (cmH_2O)	10.5 ± 1.6	9.1 ± 2.9	10.3 ± 1.7
$\Delta P,_{L}$ (cmH_2O)	7.3 ± 1.6	6.2 ± 2.9	7.2 ± 1.7
$Energy,_{L}$ (mJ)	0.95 ± 0.16	0.68 ± 0.42	0.77 ± 0.21
Power (mJ/min)	66 ± 11	47 ± 29	54 ± 15
PaO_2/FiO_2 (mmHg)	203 ± 108	236 ± 143	185 ± 84
pHa	7.38 ± 0.06	7.39 ± 0.08	7.38 ± 0.07
$PaCO_2$ (mmHg)	40 ± 8	33 ± 6	41 ± 8
HCO_3^- (mmol/L)	24 ± 3	21 ± 2	24 ± 3

Respiratory parameters and arterial blood gas analysis during mechanical ventilation in the following groups: 1) V_T = 6 mL/kg and RR adjusted to normocapnia; 2) V_T = 13 mL/kg; 3) V_T = 22 mL/kg. In the second and third groups, RR was adjusted to yield mechanical power comparable to that in the first group. Values are mean ± standard deviation (SD) of 7 animals/group. Comparisons were done using one-way ANOVA followed by Tukey's multiple comparisons ($p<0.05$). V_T: tidal volume; RR: respiratory rate; $Pplat,_{RS}$: respiratory system plateau pressure; $\Delta P,_{RS}$: respiratory system driving pressure; $Pplat,_{L}$: transpulmonary plateau pressure; $\Delta P,_{L}$: transpulmonary driving pressure; PaO_2/FiO_2: ratio of partial pressure of arterial oxygen to fraction of inspired oxygen; $PaCO_2$: partial pressure of carbon dioxide; HCO_3^-: bicarbonate.
