# Supplementary material for: Impact of Different Tidal Volume Levels at Low Mechanical Power on Ventilator-Induced Lung Injury in Rats
Source: Front Physiol. 2018 Apr 4;9:318. doi: 10.3389/fphys.2018.00318 (PMC5893648; doi:10.3389/fphys.2018.00318)
Supplement: Supplementary file 5 [file Table3.PDF]

Supplementary Material

Impact of different tidal volume levels at low mechanical power on ventilator-induced lung injury in rats

Lillian Moraes, Pedro L. Silva, Alessandra Thompson, Cintia L. Santos, Raquel S. Santos, Marcos V.S. Fernandes, Marcelo M. Morales, Vanessa Martins, Vera L. Capelozzi, Marcelo Gama de Abreu, Paolo Pelosi, Patricia R. M. Rocco*

*** Corresponding Author:** prmrocco@gmail.com

Supplementary Table 3. Coefficients of multiple linear regression.

	$Energy_{,L}$	r^2
pHa	-0.03 (-0.04, -0.02)*	0.50
$PaCO_2$ (mmHg)	4.2 (2.43, 5.89)*	0.58
PaO_2/FiO_2	-9.25 (-25.48, 6.99)	0.08
DAD score	1.71 (0.81, 2.62)*	0.47
IL-6 (fold change relative to NV)	7.1 (-2.4, 16.7)	0.19
Amphiregulin (fold change relative to NV)	0.85 (-0.73, 2.43)	0.11
CC16 (fold change relative to NV)	3.59 (1.06, 6.12)*	0.50
Decorin (fold change relative to NV)	-0.65 (-0.94, -0.36)*	0.58
Syndecan (fold change relative to NV)	0.46 (-0.03, 0.96)	0.21
MMP-9 (fold change relative to NV)	1.96 (0.61, 3.32)*	0.41

Values represent the β coefficients (95%CI) of multiple linear regression analyses. The independent variable is $Energy_{,L}$ (mechanical energy), and the dependent variables are pHa, $PaCO_2$, PaO_2/FiO_2, DAD score, IL-6, amphiregulin, CC16, decorin, syndecan, and MMP-9 gene expression. r^2 represents the percentage of the response variable variation that is explained by the linear model. * $p<0.05$.
